# Synthetic Model Combination: A new machine‐learning method for pharmacometric model ensembling

**DOI:** 10.1002/psp4.12965

**Published:** 2023-04-24

**Authors:** Alexander Chan, Richard Peck, Megan Gibbs, Mihaela van der Schaar

**Affiliations:** ^1^ Department of Applied Mathematics and Theoretical Physics University of Cambridge Cambridge UK; ^2^ Pharma Research and Development (pRED), Roche Innovation Center Basel Switzerland; ^3^ Department of Pharmacology & Therapeutics University of Liverpool Liverpool UK; ^4^ Cambridge Centre for AI in Medicine University of Cambridge Cambridge UK; ^5^ Clinical Pharmacology and Quantitative Pharmacology, Clinical Pharmacology and Safety Sciences, R&D, AstraZeneca Gaithersburg Maryland USA

## Abstract

When aiming to make predictions over targets in the pharmacological setting, a data‐focused approach aims to learn models based on a collection of labeled examples. Unfortunately, data sharing is not always possible, and this can result in many different models trained on disparate populations, leading to the natural question of how best to use and combine them when making a new prediction. Previous work has focused on global model selection or ensembling, with the result of a single final model across the feature space. Machine‐learning models perform notoriously poorly on data outside their training domain, however, due to a problem known as covariate shift, and so we argue that when ensembling models the weightings for individual instances must reflect their respective domains—in other words, models that are more likely to have seen information on that instance should have more attention paid to them. We introduce a method for such an instance‐wise ensembling of models called Synthetic Model Combination (SMC), including a novel representation learning step for handling sparse high‐dimensional domains. We demonstrate the use of SMC on an example with dosing predictions for vancomycin, although emphasize the applicability of the method to any scenario involving the use of multiple models.

**Study Highlights**

**WHAT IS THE CURRENT KNOWLEDGE ON THE TOPIC?**

Model averaging population pharmacokinetic models is known to improve the predictive accuracy when informing optimal dose selection.

**WHAT QUESTION DID THIS STUDY ADDRESS?**

Appropriately averaging models is challenging, and current methods ignore important information about the demographics of which population a model is based on, a concept this study leverages for improved performance.

**WHAT DOES THIS STUDY ADD TO OUR KNOWLEDGE?**

Incorporating demographic information into model averaging methods allows us to improve the quality of predictions while maintaining the gains of current methods.

**HOW MIGHT THIS CHANGE DRUG DISCOVERY, DEVELOPMENT, AND/OR THERAPEUTICS?**

The algorithms described in the study may increase the accuracy of precision dose calculations among other targets.

## INTRODUCTION

The ability of a model to correctly represent a population is of necessity limited by how well the data used to build it represents the population. Given the enormous variability within and between human populations for pharmacokinetics (PKs) and pharmacodynamics (PDs), this is a significant challenge for the use of models and their ability to make useful predictions about the effects in humans. This problem is further increased by the variability within diseases which are generally not homogenous entities where all patients have exactly the same disease processes.

**FIGURE 1:**
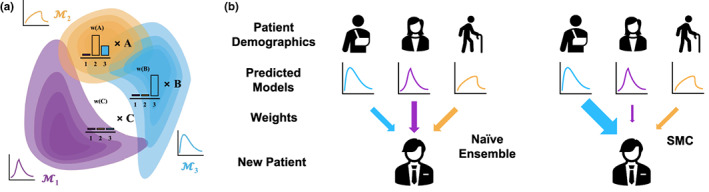
Instance‐wise ensembles. (a) Here, we represent the density of the training features for three separate models: M1, M2, and M3. Given new test points A, B, and C, we need to construct predictions from these models. A is well‐represented by both M2 and M3, whereas B only has significant density under M3. C looks like none of the models will be able to make confident predictions. (b) Different models are useful for new patients. Population pharmacokinetic models are often trained on certain demographic groups given the studies that are designed for data collection. For a new patient who does not necessarily fit into one of the existing demographics, different models may be more or less relevant and accurate. Naive ensembles ignore this fact and always incorporate evenly the predictions of each model, SMC on the other hand aims to up‐weight the models that would appear to be more relevant. SMC, Synthetic Model Combination.

When attempting to apply one or more previously developed models to make predictions about new patients or populations it is a very challenging task for us to know how well the individual models should perform, making the task of choosing the most appropriate model (or ensemble) difficult. This is compounded by the problem that the provided models could perform poorly for two main reasons. First, the model itself may not have been flexible enough to properly capture the underlying true function present in the data; and second, in the area that they are making a prediction there may not have been sufficient training data used for the model to have been able to learn appropriately (i.e., the model is extrapolating [potentially unreasonably] to cover a new feature point; Figure 1). This can be the case even when models have been designed and are based on a mechanistic understanding of a biological process, as in most cases, we still have an incomplete representation of the full process.

Various methods have been developed to address this problem. At the simplest level, there are typically multiple models developed each using different datasets that are claimed to better represent the population or at least a specific subset of the population. However, the reference populations used to build each model are usually only small subsets of the whole. Methods, such as the Bayesian model averaging, are used to combine multiple models to try to capture the value of each, however, they usually assume the model's performance is independent of the populations, ignoring their training domain. Data and model repositories have been proposed to allow the development of more definitive models of diseases and of the PK/PD of therapies.^1^ There has been some success developing publicly accessible repositories of disease models that make the data and scope of the models more transparent, for example, the Drug Disease Model Resource (DDMoRe) model repository^2^ and several academic institutions, charities, and pharma companies make some trials data available through the non‐profit organization Vivli.^3^ However, the extent of the data accessible is limited by some participants and phase I studies or PK data are excluded by some pharma participants. Sharing data brings its own challenges, not the least being that there may not be permission to use the data for anything other than the specific purpose for which it was first developed.

Synthetic Model Combination (SMC) is a new machine‐learning method that leverages and combines multiple models in an effective manner. Unlike existing methods, it focuses on building ensembles in an instance‐wise manner before any additional data have been collected, that is to say that for each new test point over which a prediction needs to be made it constructs a new ensemble. This effectively means that SMC is able to select models for each test case (patient) that it thinks will be most effective for the given case, based primarily on whether the case is likely to have been well‐represented in the training domain of each model. In this paper, we introduce and describe SMC, illustrate its use through an example of multiple pharmacometric models, and try to stimulate ideas about other possible applications relevant to pharmacometrics, clinical pharmacology, and drug development and use.

## BACKGROUND AND RELATED WORK

SMC can be used in any situation where there are multiple alternative models. For the purposes of illustrating the method, we chose to consider the common situation of multiple population PK/PD models. In the case of the antibiotic vancomycin, this is clinically important as population PK/PD models are commonly used to guide selection of doses to achieve a target area under the curve (AUC) and maximize the chances of effective therapy without nephrotoxicity and it is important to identify how best to use the multiple possible models.^4^ We emphasize that this specific example is an illustration for the purposes of explaining the methodology. We anticipate there are many other situations, including other drugs and other cases where multiple models are available for which SMC may be even more useful.

With multiple models our goal is to combine them in the most appropriate way, taking the form of constructing ensembles. Here, we differentiate between what we first describe as naive ensembling, where multiple models have been trained (through bootstrapping or on different datasets) in order to reduce the expected bias or get an estimate of distributional uncertainty. Performance‐based Model Averaging (PBMA), on the other hand, works by selecting models with higher weights based on an estimate of the performance of the model; a practical and common approach being Bayesian Model Averaging (BMA).^5^ Given an appropriate (usually uniform) prior, we calculate the posterior probability that a given model is the optimal one—and once this is obtained, the models can be marginalized out during test time predictions, creating an ensemble weighted by each model's posterior probability. The posterior being intractable, the probability is approximated using the Bayesian Information Criterion (BIC)^6^—which requires a likelihood estimate over some validation set containing labeled examples* and is estimated as:

$$\begin{array}{c} p\left(M_{i},|,D\right)=\exp\left(-\frac{1}{2}\mathrm{BIC}\left(M_{i}\right)\right)/\sum_{i=1}^{N}\exp\left(-\frac{1}{2}\mathrm{BIC}\left(M_{i}\right)\right) \end{array}$$

With this it is important to note the subtle difference in setup to the problem we are trying to work with. In all cases, it is assumed that there is some ordering for the models that hold across the feature space and so a global ensemble is produced with a fixed weighting $\hat{w}$ such that $w\left(x\right)=\hat{w}\forall x\in X$. This causes failure cases when there is variation in the models across the feature space, because it is a key point that BMA is not a model combination method.^7^ This being an important distinction and one of the main reasons BMA has been shown to perform badly under covariate shifted tasks^8^—that is to say tasks where the testing distribution differs from the training distribution, a scenario that is well known to affect the quality of a model's predictions.^9^ That being said, it can be extended by considering the set of models being averaged to be every possible combination of the provided models,^10^ although this becomes even more computationally infeasible.

This has led to a family of ensemble methods that calculate their weights slightly differently, replacing the $\mathrm{BIC}\left(M_{i}\right)$ term in the above equation with other measures of the “quality” of the model, such as Akaike information criterion, log likelihood, or negative sum of squared errors—all of which are explored in the work of Uster et al.^11^ This appears to be the extent of the exploration in model averaging in the clinical pharmacological setting. We have summarized the properties of these methods in Table 1.

**TABLE 1:** Comparison of modern model averaging/ensembling methods.

Method	Instance‐wise?	Require new data?	Weights
Naive ensemble	No	No	w=1/N
PBMA	No	Yes	$w_{i}\propto \mathrm{BIC}\left(M_{i}\right)$
Model selection	No	Yes	N/A
Synthetic Model Combination	Yes	No	$w_{i}\left(x\right)\propto p_{i}^{Z}\left(f_{\theta }\left(x\right)\right)$

Abbreviations: N/A, not applicable; PBMA, Performance‐based Model Averaging.

Traditionally PBMA models would not be used for instance‐wise predictions, because, in a typical supervised learning setting, each patient would only have a single set of covariates and outcome associated with them and so it would not be possible to get an estimate of the performance for a given individual that would be different from the population as a whole. This reflects the central problem with global ensembles that run on the assumption that the measure of “goodness” of each individual model holds the same across the feature space. That is to say that each model will be just as effective at predicting for older patients with diabetes as it would for infants, however, this is unlikely as models are often trained in different subpopulations and it is expected that they might all react differently to a drug. Global ensembles implicitly assume that this covariate shift is not the case and as such suffer when it is—they apply the same ensemble of models to every single new test point regardless of what data the model was trained on.

However, in population PK (PopPK) settings, we sometimes wish to predict a patient's AUC having already observed one or multiple observations—in this case, we would be able to use these few observations to get a performance estimate and thus weight models via PBMA. Despite this, we will likely still only have at most single digit observations for a patient and so there is a risk that we do not have enough signal to fit appropriate models and may potentially overfit. We still may want to make predictions without any observations, and as noted in ref. 11, this is an area that PBMA does not handle and simply reduces to a naive ensemble.

## METHODS

### Synthetic Model Combination

Unlike the previously mentioned methods, even without any observations at all, SMC does not search for a global ensemble—rather, it asks the question; for a given individual $x_{i}$, what do we think is the best ensemble? This could naturally vary quite considerably from individual to individual, especially if the models were trained on data from relatively disparate populations.

**FIGURE 2:**
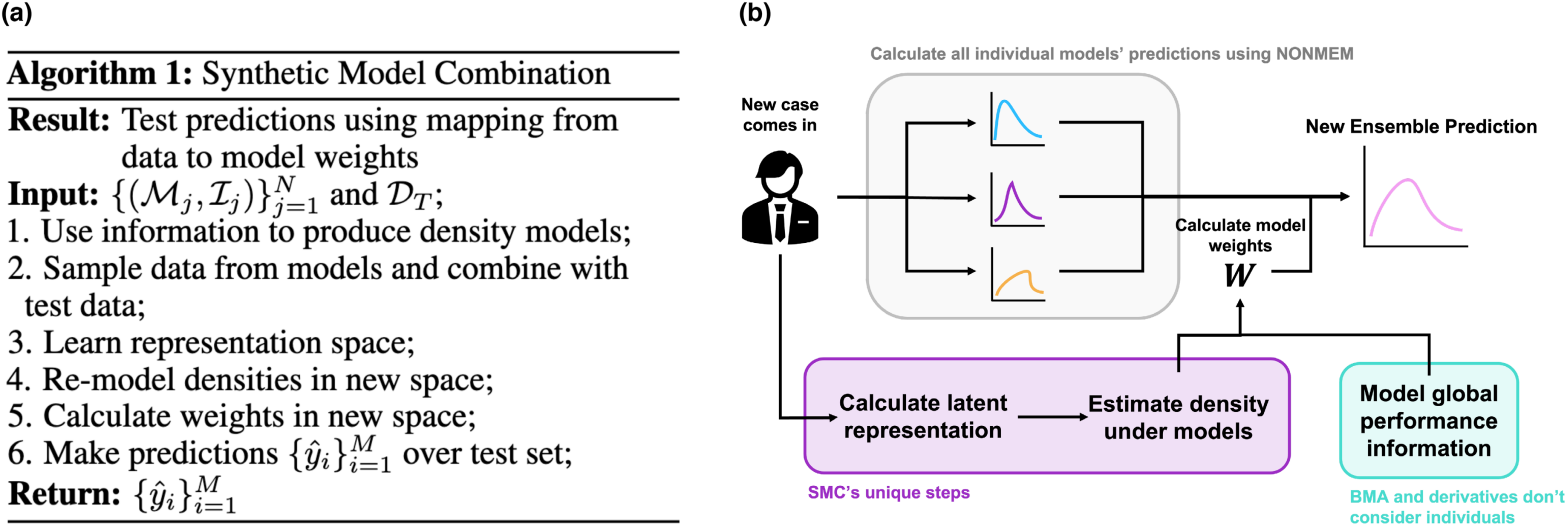
Synthetic Model Combination training algorithm. (a) Algorithm outlining the main steps in training for SMC. (b) Inference flow diagram. As a new case comes in, the first step is to calculate all of the individual models' predictions, using NONMEM, for example. Then, like with any model averaging algorithm the weights must be calculated. Performance‐based model averaging methods have a set of weights independent of the new case, whereas SMC maps the new case features to a latent space that is then used to calculate individual weights for that case. BMA, Bayesian Model Averaging; SMC, Synthetic Model Combination.

We explain at a high level the method here but refer the interested reader to ref. 12 for more in‐depth detail. The method can be broken down into essentially three main steps, an overview of which is shown in Figure 2, detailing both the algorithm for learning (Figure 2a) and inference (Figure 2b).

The first step in SMC is to use the demographic information reported alongside published models to produce a density estimate such that we can sample from each model's effective support. This aims to create a crude estimate of the region on which the model was trained on in the original feature space, for example, the general distribution of heights, weights, sex, etc. We will often expect this feature space to be high dimensional and the information available to be not hugely detailed, limiting our ability to use this original space to make meaningful predictions.

Given the flexibility in the form of what we allow the information to take, SMC must remain relatively agnostic to this step. A common example of the type of information we expect will simply be example feature samples, and, in this case, a simple kernel density estimate^13^ or other density estimation method could be used. On the other hand, when models are published, authors will often also provide demographic information on the patients that were involved in the study, such as the mean and variance of each covariate recorded. In this case, we may simply want to approximate the density using a Gaussian, and moment‐matching, for example.

The second step is to learn a representation space for the individual features which will be maximally informative for considering which models will be effective at making accurate predictions on an individual. The principal aim is to lower the relevant dimensionality of the data such that density modeling is effective in the learnt space, but this can also be effective in bending and compressing the space such that regions of model training data are moved closer together or further apart based on whether they produce useful and transferable models. In cases when the dimension of covariates is already low, this step is not always necessary. Learning the representation takes the form of a Variational / Regular Autoencoder^14^ with additional auxiliary losses. This is trained on the features of the testing set as well as samples from the densities for individual models that were generated in the first step. Choosing the latent dimension to be low results in learning a representation space that compresses the useful information in the features and aims to move training regions that are transferable closer together.

In the final step, we remodel the original densities in the feature space now in the representation space, so that we can calculate ensemble weights for individuals based on their density under each model. Given model densities in the feature space $p_{j}^{X}\left(x\right)$, we construct a corresponding density in the representation space $p_{j}^{Z}\left(z\right)$, this can be achieved simply by sampling from $p_{j}^{x}\left(z\right)$, passing through the encoder $f_{\theta }$ and modeling the new density with a kernel density estimate.

From here, we calculate weights as the relative density a feature representation has under the densities in the new space:

$$w_{i}\left(x\right)=\frac{p_{i}^{Z}\left(f_{\theta }\left(x\right)\right)+\gamma }{\sum_{j=1}^{N}p_{j}^{Z}\left(f_{\theta }\left(x\right)\right)+\gamma }$$

with a regularization hyperparameter γ chosen to be very small such that an outlier's weights are not dominated by the closest model.

This step simply weights models by an individual's density in the new representation space, meaning that models that are more likely to have seen features similar to the individual (or ones with transferable features) will play a bigger part in the ultimate prediction for the given individual. The quantity can be used to inform the confidence of any prediction made by SMC. Particularly low values will indicate that the feature had low density under all the domains and as such it may be likely that none of the models were accurate. We note as well that assuming a hierarchical generative model for the test data where one of the models training data distributions is selected and then sampled from, this can be interpreted as the posterior probability that a test instance was sampled from a model's domain and is thus well‐represented by it.

### Interacting with PBMA

The key thing to note is that, unlike PBMA methods, SMC does not need any sort of observation in order to individualize the weights of the ensembles. This means that it can be applied in areas that PBMA cannot, such as making direct a priori predictions about a patient's AUC before any observations have been observed. However, when PBMA can be applied, because both it and SMC fundamentally use different signals to generate their weights, they can reasonably be combined at the same time in order to achieve the benefits of both models (i.e., the weights from both methods can be calculated individually and then combined in order to produce a final weighting). Interestingly, in the case of BMA, we can potentially see this as a case of SMC learning an appropriate prior distribution for the weights that is then updated based on the performances of the models on the new observations, allowing for a natural integration into the current framework that allows for the best of both methods.

## RESULTS

### A case study in vancomycin

For vancomycin, the latest consensus dosing guidelines from the Infectious Diseases Society of America^15^ recommend adjusting the dose to achieve a target AUC. Many PopPK models have been developed to adjust the doses on an individual patient basis taking into account important patient covariates.

We base our experiment around those of ref. 11 who themselves consider a model averaging approach through the application of model averaging. We use simulated patients provided by the authors to evaluate the effectiveness of SMC in the accuracy of predicting the AUC across a number of settings when a number $\in \left\{0\;\left(A\;\text{priori}\right),1,2,3\right\}$ of concentration measurements are taken in a 48‐h period. Ultimately, we have six models, each from a separate subpopulation (extremely obese,^16^ critically ill post heart surgery,^17^ trauma patients,^18^ intensive care patients,^19^ septic,^20^ and hospitalized patients^21^), as well as a variety of demographic information for each. In our experiments, we focus on the age, height, weight, and creatinine clearance levels as have been shown to be strongly associated with drug response^11^ and are provided for each model.

We use the exact same test simulations as the original authors in order to more accurately explore the impact of SMC on the predictions made, we refer the interested reader to their paper for exact details of how the simulations were produced. In summary though, covariates were sampled from a global population before AUC observations being sampled for 1000 patients from each of the PopPK models used for a total cohort of 6000 simulated patients.

In Table 2, we report the relative root‐mean‐square error (RMSE) and bias of the predictions—the lower, the better. We can see that SMC consistently performs competitively, especially when combined with PBMA, although it does not appear to be outperforming the competition in any significant sense. However, we note that the simulation setup here is not based on the underlying assumption that we make (i.e., when simulating patients based on the model of Adane et al.^16^) for clinically obese patients, the current simulations still generate covariates from a normal population, and actually only a small minority of the patients would be considered obese. We believe that this is not representative of what is seen in the real world and, consequently, in order to evaluate the performance of SMC in what we consider a more realistic setting, we developed a method to subsample the original simulations in order to obtain a population for each model that more accurately reflects the population on which each model was developed.

**TABLE 2:** RMSE and bias for AUC predictions from models given number of concentration measurements over a 48‐h period on the full cohort of 6000 simulated patients.

		A priori	One	Two	Three
Bias	Adane et al.	24.7±0.3	13.3±0.1	13.3±0.1	13.3±0.1
	Mangin et al.	34.7±0.2	11.6±0.1	10.5±0.1	2.3±0.1
	Medellin‐G et al.	27.7±0.1	12.8±0.1	11.2±0.1	8.1±0.1
	Revilla et al.	12.3±0.3	1.1±0.1	0.3±0.1	2.9±0.1
	Roberts et al.	11.0±0.1	0.2±0.1	1.7±0.1	1.7±0.1
	Thomson et al.	0.5±0.1	3.1±0.1	3.7±0.1	4.0±0.1
	Naïve ensemble	10.7±0.1	6.6±0.1	6.8±0.1	5.4±0.1
	PBMA	10.7±0.1	1.1±0.1	2.2±0.1	1.7±0.0
	SMC	0.7±0.1	2.7±0.1	3.8±0.1	3.5±0.0
	SMC + PBMA	5.0±0.1	1.9±0.1	3.0±0.0	2.6±0.1
RMSE	Adane et al.	52.4±0.3	32.9±0.2	32.7±0.3	27.4±0.3
	Mangin et al.	57.4±0.2	27.8±0.1	25.5±0.3	17.7±0.1
	Medellin‐G et al.	51.4±0.2	25.7±0.1	22.8±0.1	17.3±0.1
	Revilla et al.	35.1±0.1	20.8±0.1	18.5±0.1	15.4±0.1
	Roberts et al.	31.7±0.1	18.7±0.1	18.1±0.1	14.7±0.1
	Thomson et al.	34.5±0.1	22.9±0.1	20.6±0.1	16.8±0.1
	Naïve Ensemble	38.4±0.1	21.9±0.1	20.0±0.1	16.1±0.1
	PBMA	38.4±0.1	19.0±0.1	17.4±0.1	13.9±0.1
	SMC	36.4±0.1	21.0±0.1	19.3±0.1	15.4±0.1
	SMC + PBMA	36.0±0.1	19.0±0.1	17.6±0.1	14.1±0.1

Abbreviations: AUC, area under the curve; PBMA, Performance‐based Model Averaging; RMSE, root‐mean‐square error; SMC, Synthetic Model Combination.

In order to select a smaller sample of 1000 patients, we first modeled the density of each of the patient populations based on the demographic statistics provided in each of the original papers. Then, for each of the 6000 simulated patients, we evaluated the likelihood that their covariates came from each model and selected the model with the highest likelihood. If this selected model matched the model from which the AUC observations were simulated, then the patient was kept and otherwise discarded. This mimics a rejection sampling method for the covariates from the original model demographics using the sampling method of ref. 11 as the base distribution. This results in a population where each model only simulated data for patients whose covariates were likely under their reported demographic information, a scenario that appears more reasonable as the true underlying process. In Table 3 we report the relative RMSE and bias again of the predictions, seeing a big improvement in the relative performance of SMC and its combination with PBMA.

**TABLE 3:** RMSE and bias for AUC predictions from models for given number of concentration measurements over a 48‐h period on 1000 subsampled patients.

		A priori	One	Two	Three
Bias	Adane et al.	53.4±0.5	29.9±0.4	23.8±0.3	21.1±0.2
	Mangin et al.	63.4±0.5	23.2±0.3	20.7±0.3	9.7±0.2
	Medellin‐G et al.	55.4±0.5	22.4±0.3	18.3±0.3	12.6±0.2
	Revilla et al.	3.7±0.3	4.0±0.2	3.5±0.2	4.4±0.2
	Roberts et al.	7.9±0.3	4.5±0.2	5.4±0.2	4.2±0.1
	Thomson et al.	22.6±0.4	14.2±0.3	11.3±0.3	10.7±0.2
	Naïve Ensemble	34.4±0.4	16.4±0.2	13.8±0.2	10.4±0.2
	PBMA	34.4±0.4	4.7±0.2	4.8±0.2	3.4±0.2
	SMC	18.4±0.5	8.2±0.3	7.1±0.2	5.2±0.2
	SMC + PBMA	26.4±0.1	6.5±0.2	5.9±0.2	4.3±0.2
RMSE	Adane et al.	80.0±0.7	47.3±0.4	40.9±0.6	32.5±0.3
	Mangin et al.	83.3±0.6	36.2±0.3	33.0±0.3	21.4±0.2
	Medellin‐G et al.	76.5±0.6	33.5±0.2	28.5±0.2	20.9±0.2
	Revilla et al.	32.7±0.4	21.6±0.2	20.0±0.2	16.7±0.2
	Roberts et al.	35.8±0.3	20.6±0.1	20.0±0.2	16.1±0.1
	Thomson et al.	48.2±0.4	30.2±0.2	25.8±0.2	20.9±0.1
	Naïve ensemble	55.7±0.4	28.5±0.2	24.9±0.2	19.3±0.2
	PBMA	55.7±0.4	20.8±0.2	19.3±0.2	15.2±0.2
	SMC	41.8±0.5	22.1±0.2	19.6±0.2	15.2±0.2
	SMC + PBMA	47.5±0.5	20.5±0.1	18.7±0.2	14.6±0.2

Abbreviations: AUC, area under the curve; PBMA, Performance‐based Model Averaging; RMSE, root‐mean‐square error; SMC, Synthetic Model Combination.

## DISCUSSION

It might not be immediately clear why we include these two separate results, and which one is the most appropriate to pay attention to. We refer to the first being the “original” simulation setting; and the second being an augmented population that we will now call the “realistic” setting, as we believe that this better reflects the assumptions of the real world.

Why is this more realistic? It seems clear given the diversity of models that are discovered that different models are more reflective of the underlying process in the different populations. In the “original” setting, the covariates of an individual are sampled from a global distribution, and then a random model is selected in order to simulate the drug dynamics. On the other hand, in the “realistic” setting, the covariates of an individual are again sampled from a global distribution. The difference then is that the model selected in order to simulate the drug dynamics is chosen with probability proportional to the likelihood that the individual came from each of the populations. As a concrete example, if we sample an individual with a body mass index greater than 30, then it is much more likely that we then simulate the dynamics from the model of Adane et al.^16^ rather than any of the others. Based on the previous data collected by all of the previous studies, this makes for a more realistic data generation process.

So why did we include the “original” results? Given the original simulations previous used in the most relevant case of PBMA, we thought it best to include them in order to give a fuller picture of when and how SMC can be expected to work. We think this is useful for practitioners to better understand when there might be limitations in some simulation setups.

So, what is the key takeaway? In the first set, SMC does not perform above and beyond comparative methods despite being competitive. However, this is not as realistic as the second set which we have designed to be more reflective of how we expect to see data in the real world. As such, the results on the second set are the important ones to pay attention to, and the ones for which it matters most that SMC is able to combine with PBMA and then outperform alternative methods, thus we think it should not be thought of as a limitation that SMC only outperforms on this second set, rather it should highlight the potential pitfalls of the previous, overly simplistic, simulations.

In this situation, it becomes clear that SMC can take advantage of the setting where our assumptions more appropriately line up with the simulations. Still, in the question of only using SMC or PBMA, we can see that they both seem to perform roughly equivalently. A clear and noticeable exception is in the “a priori” setting where PBMA cannot be applied properly and as such SMC significantly improves upon it. This happens because PBMA needs to see at least one extra observation in order to construct its estimate of the performance. Without it, PBMA just assumes equal performance and so weights all of the models equivalently, the same as a naïve ensemble. This is an important point to note when it comes to choosing which model one might use in practice, if we are only interested in making a priori predictions, then it is not possible to apply PBMA properly, making SMC a much‐preferred alternative that performs well. What is clearer, however, is that when both are used in combination, they can each take advantage of their different properties and outperform the other methods individually.

This highlights the separate sources of gain between SMC and PBMA, where the latter works best when a particular model just makes generally better predictions and we are able to make a good estimate of this performance. It should be pointed out as well that in cases where you have no labeled data it can be hard—or impossible—to obtain such an estimate of global performance, meaning that often this approach may not even be possible in the first place, as is pointed out in ref. 11. However, when it is possible, it does not interfere with SMC in that you can calculate weights according to both methods and then combine them as you like. The key takeaway being that by introducing SMC, you do not need to give up the benefits of global ensembling for identifying good average models.

To further understand how scenarios affect SMC, we see that raw SMC performs worse when there is high variability in the performance of individual models. Because SMC does not attempt to evaluate the relative performances of the models, when there are models that just perform very badly, they can severely detract from SMC's performance. This highlights that SMC performs best when all the models perform well in their respective domains, but that those domains are relatively disjointed. This weakness is partially offset by the combination with PBMA—this weights models globally by some level of how confident we are that the model is good as well as locally by how well we believe the model will be able to perform on a specific feature, balancing the potential causes of poor performance.

In other cases of individuals on which SMC tends to perform particularly poorly, are those which have low density in the learnt representation space under all of the models. These then become individuals for which it will then weight relatively evenly across them, this then reinforces the point of the previous paragraph, as relatively poor models are over‐represented in the proposed ensemble weights. However, these are the same individuals for which SMC has high uncertainty, for which it would recommend collecting more data, and this could be taken into account at the point of decision, opening up opportunity for SMC to highlight when it is uncertain and then decline to make a prediction, or at the least pass this uncertainty on to the practitioner.

### Applications in practice

To explain and illustrate SMC and how it differs from existing model ensembling methods, we used an example of multiple PopPK models. However, SMC is not limited to use only with Pop PK models. It can be used in any situation where multiple models have been developed to address the same issue, for example, multiple disease progression models of the same disease. It is likely there are other situations too and we hope to inspire ideas for other applications.

In order to identify good models on the individual level, SMC models the regions of the feature space for which models should be able to produce good predictions based on a level of epistemic uncertainty. This epistemic uncertainty could in turn be used in a feedback system for identifying regions of the space for which we have no good models. This would allow for future targeted data collection, allowing practitioners to identify subpopulations that require more information, not wasting resources collecting information on patients which we can already predict well for.

**FIGURE 3:**
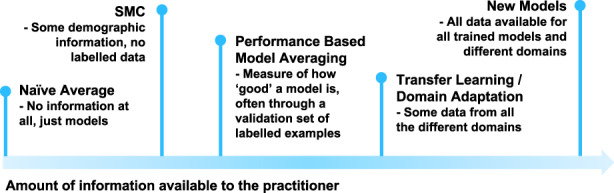
Methods based on varying information. A selection of methods from the spectrum of information available to a practitioner. SMC lies quite far toward the little information end, aiming to only take use of some demographic information from each of the models and not require any labeled training points. SMC, Synthetic Model Combination.

As we show in Figure 3, the machine‐learning community has developed a range of methods for what to do in the cases of different amounts of information. SMC is not the definitive answer on how to use the knowledge of model training domains, but it is the first, and highlights an important consideration that practitioners should be aware of when making their own predictions. We hope that this work will inspire future investigation, particularly in the task of adapting models to new populations we have no existing data on.

In general, we aim for the key takeaway for practitioners to be the following: *If the individual models seem unlikely to transfer well across populations, then incorporating an aspect of SMC into ensemble predictions is unlikely to damage predictive power in the worst case and will most likely improve predictions*.

To conclude, in this paper, we have introduced the framework of SMC to the clinical pharmacological and pharmacometric community—an instance‐wise approach to ensembling models in order to make predictions with only models that have seen similar individuals during their training phase. We demonstrated how it can be applied in the averaging of PopPK models with the real case study of estimating the effectiveness of vancomycin precision dosing, and the impact that could have in terms of the appropriate treatment of patients.

## AUTHOR CONTRIBUTIONS

A.C., R.P., M.G., and M.V.S. wrote the manuscript. A.C. and M.V.S. designed the research. A.C. performed the research. A.C. and R.P. analyzed the data.

## FUNDING INFORMATION

No funding was received for this work.

## CONFLICT OF INTEREST STATEMENT

The authors declared no competing interests for this work.

## Endnote

* By labeled examples we mean covariate‐target pairs {(x,y)} ‐ whereas unlabeled examples only contain the covariates {x}.
